# Resveratrol Alleviates Effects of LPS on Estrogen Synthesis, Oxidative Stress, Inflammation, and Pyroptosis of Goat Granulosa Cells by Activating the PPARG/NRF2/HO-1 Signaling Pathway

**DOI:** 10.3390/antiox14111300

**Published:** 2025-10-29

**Authors:** Jie Zhao, Xianyi Zhou, Zhen Cang, Xin Liu, Muhammad Tariq, Dagan Mao

**Affiliations:** College of Animal Science and Technology, Nanjing Agricultural University, Nanjing 210095, China; 2021205014@stu.njau.edu.cn (J.Z.); zhouxianyi@stu.njau.edu.cn (X.Z.); cangzhen@stu.njau.edu.cn (Z.C.); 2025105025@stu.njau.edu.cn (X.L.); tariq@stu.njau.edu.cn (M.T.)

**Keywords:** resveratrol, lipopolysaccharide, granulosa cells, PPARG/NRF2/HO-1 signaling pathway, ovary inflammation

## Abstract

This study aims to investigate the effect and mechanism of resveratrol (RES) on lipopolysaccharide (LPS)-induced injury in goat granulosa cells (GCs). First, the appropriate time and concentration were screened for LPS (4 μg/mL, 12 h), RES (1 μM, 6 h), and GW9662 (an antagonist of PPARG, 1 μM, 12 h) through CCK8 and RT-qPCR. Then, cells were treated with LPS, RES, or/and GW9662, to examine steroidogenesis, inflammation, oxidative stress, and pyroptosis by RIA, RT-qPCR, WB, flow cytometry, and IF, respectively. Results showed that RES inhibited LPS-induced increases in MDA content, ROS production, gene expressions of IL-1β, NLRP3, Caspase1, and GSDMD, as well as protein levels of IL-1β, and GSDMD, accompanied by decreases in SOD activity, T-AOC and E2 content, gene expressions of SOD, CYP19A1, and HSD3B, and protein levels of SOD and HSD3B. Furthermore, RES inhibited LPS-induced decreases in PPARG, NRF2, and HO-1 gene expressions and protein levels. However, GW9662 could block all the alleviating effects of RES on LPS. In conclusion, RES regulates the effects of LPS on hormone secretion, inflammation, oxidative stress, and pyroptosis by modulating the PPARG/NRF2/HO-1 pathway, providing a new theoretical basis for improving goat reproduction.

## 1. Introduction

Oxidative stress and inflammation are the major factors that influence the functioning of the ovaries, the growth of follicles, and the quality of oocytes. Overproduction of reactive oxygen species (ROS) disrupts granulosa cell activity and steroidogenesis, resulting in reduced fertility in animals [1,2], also activates the TLR4/NF-κB signaling pathway and increases the levels of pro-inflammatory cytokines such as IL-1, IL-6, and TNF-α. The bacteria endotoxin lipopolysaccharide (LPS) stimulates oxidative stress and apoptosis in ovarian cells [3]. The effects of these inflammations disrupt hormone production and follicular development, and protective measures must be implemented to ensure that ovaries stay healthy.

Oxidative stress and inflammation have also been cited as major contributors to ovarian dysfunction and infertility in humans [4]. The uncontrolled production of reactive oxygen species (ROS) and inflammatory cytokines interferes with steroidogenesis, oocyte maturation, and follicular development, which relate to such reproductive disorders as polycystic ovary syndrome (PCOS), endometriosis, and premature ovarian failure [5]. Oxidative stress is a crucial factor affecting female reproduction in humans, as it impairs oocyte quality and disrupts embryo development. Oxidative imbalance was a determinant pathophysiology in human ovaries [6]. Inflammatory damage to granulosa cells due to (LPS) is mediated by the amplification of the TLR4/NF-KB pathway and the reduction in estrogen [7]. Therefore, the ovarian dynamics and future approaches involving antioxidation and anti-inflammatory effects may be comprehended by a more insightful evaluation of the antioxidant and anti-inflammatory effects on humans.

LPS affects the expression of the antioxidant enzyme (superoxide dismutase, SOD) and alters the cellular functional and metabolic status [8,9]. LPS causes cells to produce large amounts of inflammatory factors, such as tumor necrosis factor-α (TNF-α), interleukin-1β (IL-1β), and interleukin-6 (IL-6) [10], resulting in inflammation and oxidative stress. LPS can also trigger the expressions of cysteine–aspartic acid protease 1 (Caspase1), nucleotide-binding oligomerization of domain-like receptor protein 3 (NLRP3), and gasdermin D (GSDMD) [11], which leads to pyroptosis, thus affecting the normal functions of the organism.

Resveratrol (RES) is a natural polyphenol that is widely found in grapes, red wine, and peanuts [12], with various functions, such as antioxidants, anti-inflammatory, anti-aging, and anti-obesity [13]. RES can scavenge or inhibit the generation of free radicals, thus protecting cells from oxidative damage [14], suppressing inflammatory responses, and alleviating tissue damage. It can also enhance immune activity by strengthening the immune system and restoring the impaired immune function [15]. Peroxisome proliferator-activated receptor (PPAR) is a ligand-activated nuclear receptor family that includes three subtypes: A, D, and G. Among them, PPARG is expressed in a variety of tissues, such as the brain [16], ovaries [17], liver [18] and lung [19], playing important roles in cell proliferation [20], apoptosis [21], and inflammation [22].

Despite the growing evidence of resveratrol’s antioxidant effects, its precise regulatory mechanisms in ruminant ovarian cells, particularly in goats, remain poorly defined. Studies have shown that RES can act as a ligand activator of PPARG to exert its transcriptional regulatory function [23], and RES and PPARG have a synergistic effect in the regulation of the inflammatory signaling pathway [24,25], which leads to the inhibition of pro-inflammatory cytokines and oxidative damage in various cells [23,24]. In addition, RES activates the nuclear factor erythroid 2-related factor 2 (NRF2)/heme oxygenase-1 (HO-1) signaling pathway that protects the cell against oxidative stress and inflammation [26]. RES reduced the expression of inflammatory mediators in the microglial murine cell line BV-2 [27], enhanced brain defenses against acute LPS stimuli in aged animals [28], and prevented palmitate-induced IL-6 and TNF-α in C2C12 cells [29]. RES can also reverse the fluoride-induced decrease in PPARG in the rat ameloblast cell line LS8 [30] and attenuate pyroptotic-driven damage in the rat retina and MGC by inhibiting the NLRP3/GSDMD/Caspase1 pathway [30]. Furthermore, rosiglitazone, an agonist of PPARG, upregulated NRF2 in a concentration-dependent manner, leading to the activation of the downstream HO-1, which plays an important role in reducing inflammation [31].

Numerous studies have shown that subacute ruminal acidosis increased serum LPS level in ruminants [32,33]. Other factors like the hygiene of forage and drinking water, or diseases, could also produce large amounts of LPS in goats, which may induce ovarian inflammation, affecting both follicle development function and luteal function. Our previous studies have shown that feeding with a high-concentrate diet leads to an increase in the serum LPS of lambs, LPS affects the proliferation and hormone secretion in luteinized granulosa cells (GCs) [34], and PPARG affects the proliferation, apoptosis, and estrogen secretion in goat GCs [35].

Therefore, the purpose of the study was to examine the protective role and mechanisms of resveratrol (RES) against lipopolysaccharide (LPS)-induced oxidative stress, inflammation, pyroptosis, and disrupting the production of estrogens in goat granulosa cells (GCs). We hypothesized that RES alleviates the effect of LPS on estrogen synthesis, oxidative stress, inflammation, and pyroptosis of GCs by activating the PPARG/NRF2/HO-1 signaling pathway. In the current study, the proper concentration and time for LPS, RES, or GW9662 (an antagonist of PPARG) treatment in goat GCs were screened first by cell-counting kit-8 (CCK8) and a real-time quantitative polymerase chain reaction (RT-qPCR) to establish an inflammatory model. Then, GCs were pretreated with or without RES or/and GW9662, followed by LPS exposure, to investigate oxidative stress, inflammation, pyroptosis, E2 synthesis, and the PPARG/NRF2/HO-1 pathway by radioimmunoassay (RIA), flow cytometry, immunofluorescence (IF), RT-qPCR, and Western blot (WB), respectively, which will uncover the underlying possible mechanisms in which RES alleviates the effect of LPS in goat GCs, and provide a new strategy and insight for the treatment of reproductive disorders.

## 2. Materials and Methods

### 2.1. Cell Isolation and Culture

Goat ovaries were obtained at a slaughterhouse (Danyang, Jiangsu), placed in saline (30–35 °C, including 100 IU/mL penicillin and 100 mg/mL streptomycin), and brought back to the laboratory within 2 h for GCs isolation, as described previously [35]. Briefly, GCs from healthy follicles (2–5 mm) were washed with Phosphate-Buffered Saline (PBS) 3 times, then transferred to T-75 culture flasks with cell medium (DMEM-F12 supplemented with 10% fetal bovine serum, 100 IU/mL of penicillin, and 100 μg/mL of streptomycin). Cells were cultured in a 37 °C humidified cell incubator with 5% CO_2_.

### 2.2. Experimental Design

In the pre-experiment, cells were treated with LPS (0–16 μg/mL for 12 h), RES (0–10 μM for 12 h and 1 μM for 0–24 h), or GW9662 (0–20 μM for 12 h and 1 μM for 0–24 h) to detect GCs viability by CCK8 assay. The preliminary data and the literature on granulosa cells in goats and other animals were used to choose the initial range of LPS concentrations (0–16 μg/mL) and treatment times (0–24 h) [36,37]. The oxidative status and ROS content of GCs exposed to LPS were also detected by commercial kits and flow cytometry, respectively. Then, GCs were exposed to the selected concentrations and treatment durations of LPS, RES, and GW9662 to confirm the optimal experimental conditions by evaluating cell viability and the expression of genes related to inflammation and pyroptosis. The optimal condition was determined as 4 μg/mL at 12 h of inducing a reproducible inflammatory response in goat GCs. The 6 h RES pretreatment period was selected because it allowed sufficient activation of antioxidant signaling pathways before LPS stimulation, consistent with the results of the pre-experiment and previous studies.

Lipopolysaccharide (LPS, from Escherichia coli 055: B5, ≥99% purity; Sigma-Aldrich, L2880, St. Louis, MO, USA) was dissolved in sterile Phosphate-Buffered Saline (PBS) to prepare stock solutions. Resveratrol (RES, ≥98% purity; Selleck Chemicals, S1396, Houston, TX, USA) and GW9662 (≥98% purity; Selleck Chemicals, S2915, Houston, TX, USA), a selective PPARG antagonist, were dissolved in dimethyl sulfoxide (DMSO) to prepare 10 mM stock solutions. These were diluted with culture medium to the shown working concentrations (LPS 4 μg/mL, RES 1 μM, GW9662 1 μM). The final DMSO concentration in all treatments was <0.1%, which did not affect cell viability.

To explore whether RES alleviates the effects of LPS through PPARG, cells were divided into four groups: control (CON), LPS (4 μg/mL for 12 h), RES + LPS, and GW9662 + RES + LPS groups. Culture media and cells were collected for subsequent analyses of oxidative indexes, E2 levels, gene and protein expression, and ROS content, as described below.

### 2.3. Cell Viability

Cell viability was analyzed using CCK8 assay (NCM Biotech, Suzhou, Nanjing, China) according to the manufacturer’s instructions [35]. Cells were treated with specific drugs in a 96-well plate, and six replicate wells were set. After treatment, the cells were incubated with CCK-8 working solution for 2 h at 37 °C in a humidified incubator containing 5% CO_2_, and the absorbance was then measured at 450 nm (Thermo Fisher Scientific, Waltham, MA, USA).

### 2.4. Radioimmunoassay

A commercial RIA kit (Beijing North Institute of Biological Technology, Beijing, China) was used to detect E2 levels in 6-well plate media at Shanghai Xinfan Biotechnology Co., Ltd., Shanghai, China. The assay had a sensitivity of 0.02 ng/mL. The intra- and inter-assay coefficients of variation (CV) were <10% and <15%, respectively. E2 concentrations were calculated from a standard curve and reported as (ng/mL).

### 2.5. Measurement of the Oxidative Indexes

GCs were seeded in a 6-well plate (5 × 10^5^ cells/well) and treated as mentioned above. The total antioxidant capacity (T-AOC, G0115W) level, superoxide dismutase (SOD, G0101W) activity, and malondialdehyde (MDA, G0109W) content in the media were analyzed using commercial kits (Suzhou Geruisi Biotechnology, Suzhou, China), according to the manufacturer’s instructions. All biochemical parameters, including enzyme activities and hormone concentrations, were expressed in (U/mL) or (ng/mL), following the manufacturer’s instructions for each commercial assay kit.

### 2.6. Detection of ROS

#### 2.6.1. Flow Cytometry Analysis

GCs were seeded into 6 well-plates and treated as mentioned above. Cells were then washed 3 times by PBS for 5 min each time and centrifuged at 1000 rpm for 5 min to adjust the density to 10^6^/mL. The intracellular ROS content was measured using a commercial kit (KGT010-1, Jiangsu KGI Biotechnology Co., Ltd., Nanjing, China). Briefly, cells were washed 3 times in serum-free medium, and then incubated with diluted DCFH-DA (10 µM) at 37 °C for 30 min, followed by washing to remove redundant DCFH-DA. The fluorescein isothiocyanate detection was performed by a BD FACSVerse™273 flow cytometer (BD Biosciences, Franklin Lakes, NJ, USA), with an excitation wavelength of 488 nm and an emission wavelength of 530 nm. The quantification was performed using ImageJ software (version 1.53, National Institutes of Health, Bethesda, MD, USA).

#### 2.6.2. Fluorescent Staining

GCs were seeded onto cover slips and cultured in a 6-well plate, as mentioned above. After treatment for a specific time, an appropriate volume of diluted DCFH-DA (10 µM) was added. Cells were incubated in the dark at 37 °C for 30 min. Cells were then washed with serum-free culture medium to remove redundant DCFH-DA. Images were captured using a fluorescence microscope (Nikon, Tokyo, Japan). The quantification was performed using ImageJ software (version 1.53, National Institutes of Health, Bethesda, MD, USA).

### 2.7. Real-Time Quantitative PCR Analysis

RT-qPCR was performed as previously described [38]. GCs were seeded in a 6-well plate and treated as mentioned above. Total RNA was isolated using RNA isolated extraction reagent (R401-01, Vazyme Biotech Co., Ltd., Nanjing, China), and reverse-transcribed to cDNA using the HiScript III RT SuperMix for qPCR (R323-01, Vazyme Biotech Co., Ltd., Nanjing, China), according to the manufacturer’s protocol. Each 20 μL of the PCR reaction was prepared as follows: 1 μL cDNA, 10 μL SYBR master mix, 8.2 μL nuclease-free water, and 0.4 μL each of forward and reverse primer pairs (10 μmol). PCR was conducted on an ABI 7300 Fast Real-time PCR System (Applied Biosystems, Foster City, CA, USA) using ChamQ Universal SYBR qPCR Master Mix (Q711, Vazyme Biotech Co., Ltd., Nanjing, China). The qPCR running system included a hold period at 95 °C for 5 min, followed by 40 cycles of 95 °C for 10 s, 60 °C for 30 s, 95 °C for 15 s, 60 °C for 1 min, and 95 °C for 15 s. RT-qPCR results were normalized to the reference gene glyceraldehyde 3-phosphate dehydrogenase (*GAPDH*). The relative expression levels of target genes were calculated based on the threshold cycle (Ct) values, using the comparative 2^−ΔΔCt^ formula. Each sample represented one biological replicate derived from granulosa cells that were isolated from three different goats, and each treatment was analyzed in triplicate wells. The sequences of the target gene were shown in Table 1.

### 2.8. Western Blot Analysis

WB was performed as previously described [39]. GCs were seeded in a 6-well plate and treated as mentioned above. Total protein was extracted with radioimmunoprecipitation assay lysate (Biosharp Life Sciences, Hefei, China), including phenylmethanesulfonylfluoride (Biosharp Life Sciences, Hefei, China). The protein concentration was detected by a bicinchoninic acid assay kit (Beyotime, Shanghai, China). Protein was separated by 10% SDS–polyacrylamide gels and transferred to polyvinylidene fluoride membranes (Millipore; Billerica, MA, USA) at 4 °C. Membranes were blocked with 5% nonfat milk for 1.5 h at room temperature, followed by washing and incubation with specific antibodies at 4 °C overnight (Table 2). After washing with TBST 3 times, the secondary antibody was incubated at room temperature for 2 h. The bands were detected using a chemiluminescence detection system (Fujifilm, Tokyo, Japan, and quantified using ImageJ software (National Institutes of Health, Bethesda, MD, USA). Cells from the untreated control (CON) group were used as the reference sample, and β-actin served as the internal loading control for the normalization of protein expression.

### 2.9. Immunofluorescence Analysis

IF was performed as previously described [40]. GCs were seeded onto cover slips in 6-well plates and treated as mentioned above. After treatment for a specific time, cells were fixed in 4% paraformaldehyde (Beyotime Biotech, Shanghai, China) for 12 h and blocked with 5% bovine serum albumin (Solarbio, Beijing, China). The cells were then incubated overnight at 4 °C with primary antibodies against Caspase1 or GSDMD (1:200 dilution), and subsequently with 594-conjugated donkey anti-rabbit secondary antibody (1:500 dilution, Bioss, Beijing, China) for 1 h at room temperature in the dark. Nuclei were counterstained with DAPI (1 μg/mL for 5 min, Solarbio, Beijing, China). The negative control was incubated with PBS instead of the primary antibody. Images were captured using a fluorescence microscope (Nikon, Japan) and quantified using ImageJ software (National Institutes of Health, MD, USA).

### 2.10. Statistical Analysis

All experiments were performed independently, using GCs, which are isolated from at least three different goats (*n* = 3 biological replicates), with each treatment conducted in at least triplicate wells (technical replicates). Data are expressed as mean ± SEM to reflect experimental reproducibility. The *t*-test was used to evaluate the significance between the two groups. Statistical differences were considered to be significant at *p* < 0.05.

## 3. Results

### 3.1. Effect of Different LPS Concentrations and Times on Goat GCs

**Figure 1:**
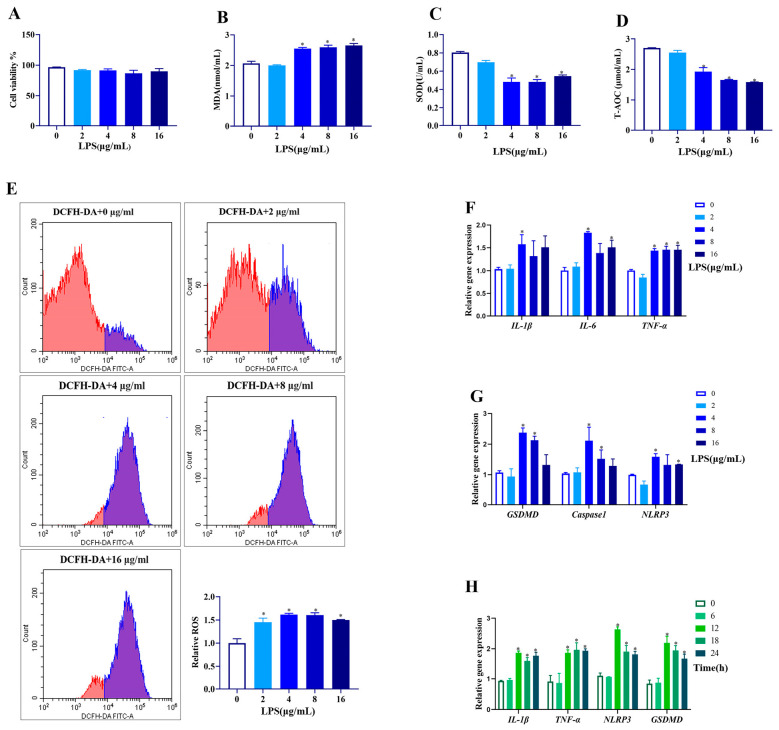
Effect of different concentrations and times of LPS treatment on cell viability, antioxidative status, expressions of genes related to inflammation, and pyroptosis in goat GCs. (**A**–**G**) Cells were exposed to 0–16 μg/mL LPS for 12 h: (**A**) cell viability; (**B**) MDA content; (**C**) SOD activity; (**D**) T-AOC content; (**E**) ROS content detected by flow cytometer and quantitative analysis; (**F**) relative mRNA abundances of *IL-1β*, *IL-6*, and *TNF-α*; and (**G**) relative mRNA abundances of *GSDMD*, Caspase1, and *NLRP3*. (**H**) Cells were exposed to 4 μg/mL LPS for 0–24 h, and relative mRNA abundances of *IL-1β*, *TNF-α*, *NLRP3*, and GSDMD were detected. All data are presented as the mean ± SEM. * indicates *p* < 0.05 compared with CON group.

To establish an inflammatory model of goat GCs, cells were first exposed to different concentrations of LPS for 12 h. As shown in Figure 1A, different concentrations of LPS (2, 4, 8, 16 μg/mL) had no effect on the viability of GCs (*p* > 0.05). However, LPS at 4–16 μg/mL increased the MDA content (*p* < 0.05, Figure 1B), and reduced SOD activity and T-AOC content (*p* < 0.05, Figure 1C,D). Different concentrations (2–16 μg/mL) of LPS significantly increased ROS production (*p* < 0.05, Figure 1E). Furthermore, LPS at 4 μg/mL significantly increased all the mRNA abundances of *IL-1β*, *IL-6*, and *TNF-α* (Figure 1F), as well as *GSDMD*, *Caspase1*, and *NLRP3* (*p* < 0.05, Figure 1G).

Regarding the time effect (0, 6, 12, 18, and 24 h), LPS treatment for 12–24 h at 4 μg/mL upregulated the mRNA abundances of *IL-1β*, *IL-6*, *GSDMD*, and *NLRP3* (*p* < 0.05, Figure 1H). Therefore, the LPS treatment at 4 μg/mL for 12 h was applied for further experiments.

### 3.2. Effect of Different RES Concentrations and Times on Goat GCs

**Figure 2:**
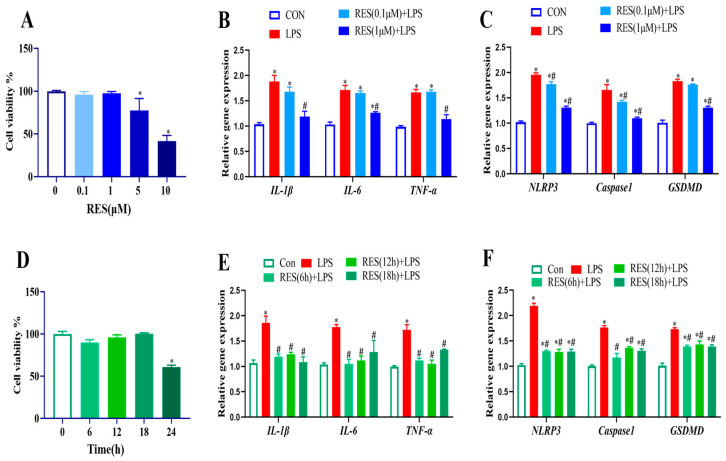
Effect of different concentrations and times of RES treatment on cell viability, expressions of genes related to inflammation, and pyroptosis in goat GCs. (**A**) Cells were exposed to 0–10 μM RES for 12 h, and cell viability was detected; (**B**,**C**) cells were pretreated with 0.1, 1 μM Res for 12 h, followed by exposure to 4 μg/mL LPS for 12 h, and relative mRNA abundances of *IL-1β*, *IL-6*, and *TNF-α* (**B**) as well as *NLRP3*, *Caspase1*, and *GSDMD* (**C**) were detected; (**D**) cells were exposed to 1 μM RES for 0–24 h and cell viability was detected; (**E**,**F**) cells were pretreated with 1 μM Res for 6, 12, 18 h followed by exposure to 4 μg/mL LPS for 12 h, relative mRNA abundances of *IL-1β*, *IL-6*, and *TNF-α* (**E**) as well as *NLRP3*, *Caspase1*, and *GSDMD* (**F**) were detected. All data are presented as the mean ± SEM. * indicates *p* < 0.05 compared with CON group. # indicates *p* < 0.05 compared with LPS group.

To optimize the concentration and treatment time of RES alleviating the LPS effect, GCs were first treated with RES (0, 0.1, 1, 5, and 10 μM) for 12 h, and results showed that RES at 5 or 10 μM significantly reduced the cell viability (*p* < 0.05, Figure 2A). Therefore, cells were pretreated with RES at 0.1 or 1 μM, followed by LPS exposure at 4 μg/mL for 12 h to detect the expression of genes related to inflammation and pyroptosis. LPS-induced gene expression levels of *IL-1β*, *IL-6*, *TNF-α*, *GSDMD*, *Caspase1*, and *NLRP3* were all significantly reduced by pretreatment with 1 μM RES (*p* < 0.05, Figure 2B,C).

Similarly, different pretreatment times (0, 6, 12, 18, and 24 h) were set with 1 μM RES., and results showed that treatment with RES for 24 h significantly reduced cell viability (*p* < 0.05, Figure 2D). Therefore, cells were pretreated with RES for 6–18 h, followed by exposure to LPS, and results showed that LPS exposure increased the gene expression levels of *IL-1β*, *IL-6*, *TNF-α*, *GSDMD*, *Caspase1*, and *NLRP3*, which were all significantly reduced by pretreatment with 1 μM RES for 6 h (*p* < 0.05, Figure 2E,F). Hence, the RES treatment at 1 μM for 6 h was applied for the further experiment.

### 3.3. Effect of Different GW9662 Concentrations and Times on Goat GCs

**Figure 3:**
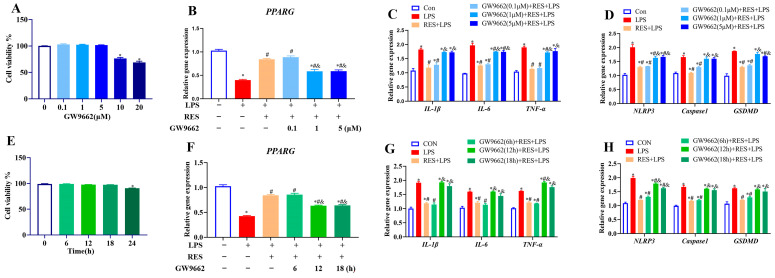
Effect of different concentrations and times of GW9662 treatment on cell viability, PPARG expression, and genes related to inflammation and pyroptosis in goat GCs. (**A**) Cells were exposed to 0–20 μM GW9662 for 12 h and cell viability was detected; (**B**–**D**) cells were exposed to 0.1, 1, 5 μM GW9662 for 12 h, followed by 1 μM Res for 6 h and 4 μg/mL LPS for 12 h, relative mRNA abundances of *PPARG* (**B**), *IL-1β*, *IL-6*, and *TNF-α* (**C**), as well as NLRP3, Caspase1, and GSDMD (**D**) were detected; (**E**) cells were exposed to 1 μM GW9662 for 0–24 h and cell viability was detected; (**F**–**H**) cells were pretreated with to 1 μM GW9662 for 6, 12, 18 h, followed by 1 μM Res for 6 h and 4 μg/mL LPS for 12 h, relative mRNA abundances of *PPARG* (**F**), *IL-1β*, *IL-6*, and *TNF-α* (**G**), as well as *NLRP3*, *Caspase1*, and *GSDMD* (**H**) were detected. All data are presented as the mean ± SEM. * indicates *p* < 0.05 compared with CON group. # indicates *p* < 0.05 compared with LPS group. & indicates *p* < 0.05 compared with RES + LPS group.

To optimize the concentration and treatment time of GW9662 to block the effect of RES on LPS, GCs were first treated with GW9662, ranging from 0 to 20 μM for 12 h. The results showed that 10 or 20 μM GW9662 significantly decreased cell viability (*p* < 0.05, Figure 3A). Therefore, cells were pretreated with GW9662 at concentrations of 0.1, 1, and 5 μM for 12 h, followed by the addition of RES (1 μM for 6 h) or/and LPS (4 μg/mL for 12 h) to detect the expression of *PPARG*, and genes related to inflammation and pyroptosis. Results showed that RES reversed the LPS-induced decrease in *PPARG* expression, which was blocked by GW9662 (1 or 5 μM) (*p* < 0.05, Figure 3B). RES inhibited LPS-induced increases in the expression of *IL-1β*, *IL-6*, *TNF-α*, *NLRP3*, *Caspase1*, and *GSDMD*, which were all reversed by GW9662 (1 or 5 μM) (*p* < 0.05, Figure 3C,D). Therefore, a concentration of 1 μM was selected for time screening, based on the results.

Then, GCs were treated with 1 μM GW9662 for 0 to 24 h, and GW9662 treatment for 24 h decreased cell viability (*p* < 0.05, Figure 3E). Therefore, cells were pretreated with GW9662 at 1 μM for 6, 12, and 18 h, followed by exposure to RES (1 μM for 6 h) or/and LPS (4 μg/mL for 12 h). Results showed that RES reversed the LPS-induced decrease in *PPARG* expression (*p* < 0.05, Figure 3F) and increases in the expressions of *IL-1β*, *IL-6*, *TNF-α*, *NLRP3*, *Caspase1*, and *GSDMD*, all of which were blocked by GW9662 for 12 or 18 h (*p* < 0.05, Figure 3G,H). Therefore, the 12 h of GW9662 treatment at 1 μM was chosen in further experiments.

### 3.4. Resveratrol Alleviates the Effect of LPS on Oxidative Stress in Goat GCs by Activating PPARG

**Figure 4:**
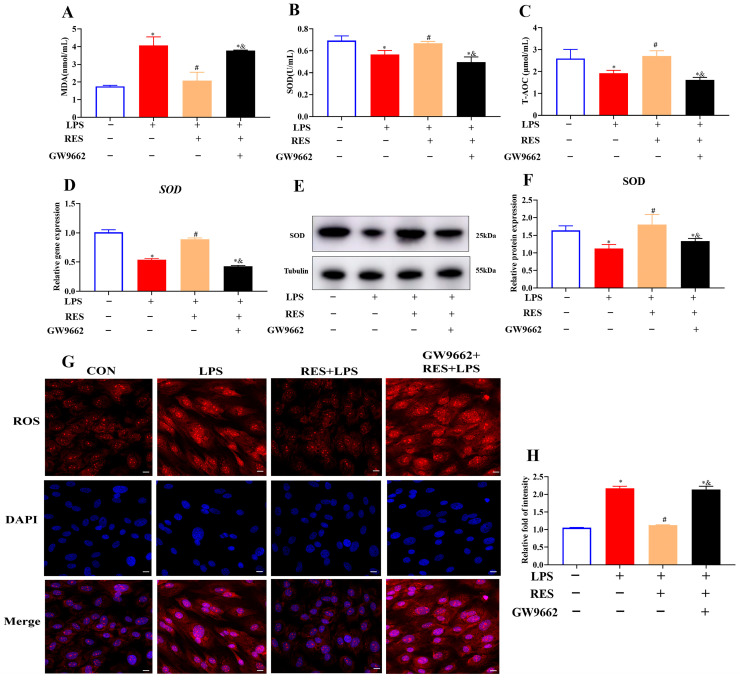
Resveratrol alleviates LPS-induced oxidative stress by activating PPARG in goat GCs. Cells were treated with or without GW9662 (1 μM for 12 h), RES (1 μM for 6 h), or LPS (4 μg/mL for 12 h). (**A**) MDA content; (**B**) SOD activity; (**C**) T-AOC content; (**D**) relative mRNA abundances of SOD; (**E**) representative protein bands of SOD; (**F**) relative protein abundances of SOD; (**G**) representative immunofluorescence images of ROS, scale bar = 20 μm; (**H**) quantitative analysis of ROS immunofluorescence intensity. All data are presented as the mean ± SEM. * indicates *p* < 0.05 compared with CON group. # indicates *p* < 0.05 compared with LPS group. & indicates *p* < 0.05 compared with RES + LPS group.

To investigate whether RES alleviates the effect of LPS on the oxidative stress of goat GCs through PPARG, GCs were assigned to four groups to detect the oxidative indexes. Results showed that RES significantly decreased the LPS-induced increase in the MDA content (*p* < 0.05, Figure 4A), and reversed the LPS-induced decreases in the SOD activity and T-AOC content (*p* < 0.05, Figure 4B,C). Compared with the LPS group, RES pretreatment significantly increased the gene and protein expression levels of SOD (*p* < 0.05, Figure 4D–F) and reduced the fluorescence intensity of ROS (*p* < 0.05, Figure 4G,H). GW9662 reversed these effects, suggesting that the effects of RES were PPARG-dependent.

### 3.5. Resveratrol Alleviates the Effect of LPS on Inflammation and Pyroptosis in Goat GCs by Activating PPARG

**Figure 5:**
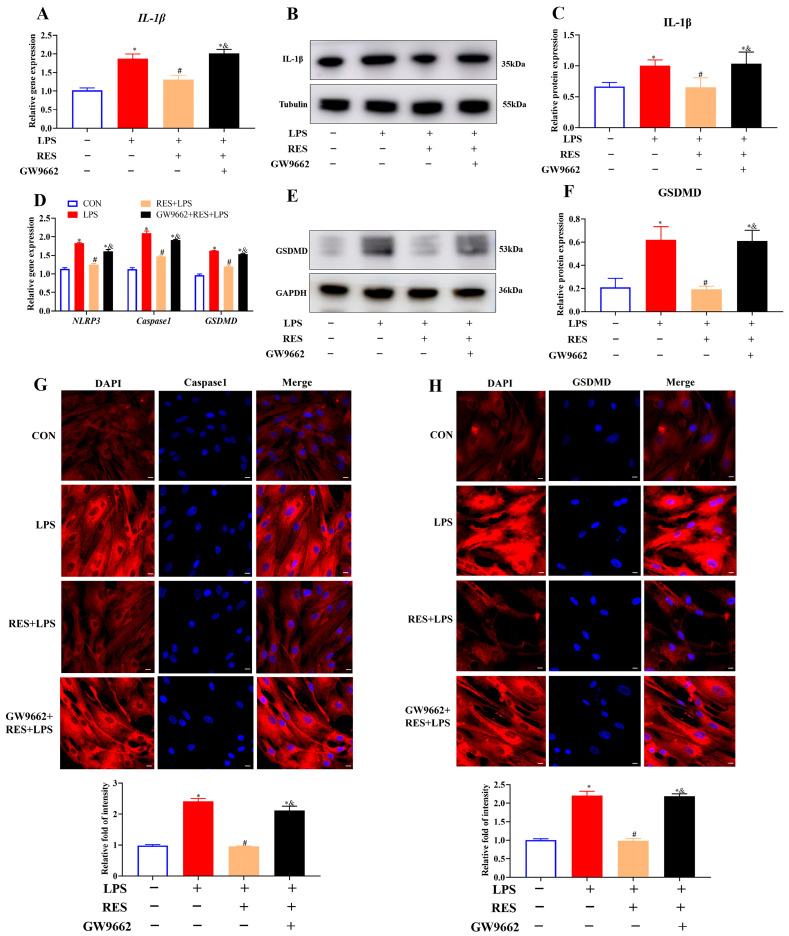
Resveratrol alleviates LPS-induced inflammation and pyroptosis by activating PPARG in goat GCs. Cells were treated with or without GW9662 (1 μM for 12 h), RES (1 μM for 6 h), or LPS (4 μg/mL for 12 h). (**A**) Relative mRNA abundances of *IL-1β*; (**B**) representative protein bands of IL-1β; (**C**) relative protein abundances of IL-1β; (**D**) relative mRNA abundances of *NLRP3*, *Caspase1*, and *GSDMD*; (**E**) representative protein bands of GSDMD; (**F**) relative protein abundances of GSDMD; (**G**) representative fluorescence images of Caspase1 and quantitative analysis, scale bar = 20 μm; (**H)** representative fluorescence images of GSDMD and quantitative analysis, scale bar = 20 μm. All data are presented as the mean ± SEM. * indicates *p* < 0.05 compared with CON group. # indicates *p* < 0.05 compared with LPS group. & indicates *p* < 0.05 compared with RES + LPS group.

To investigate whether RES alleviates the effects of LPS on inflammation and pyroptosis in goat GCs through PPARG, GCs were assigned to four groups to detect the expression of related genes and proteins. The results showed that RES pretreatment significantly decreased the LPS-induced gene and protein expressions of IL-1β (*p* < 0.05, Figure 5A–C), gene expression levels of *NLRP3*, *Caspase1*, and *GSDMD*, as well as the protein level of GSDMD (*p* < 0.05, Figure 5D–F). However, all these effects were reversed by GW9662, which is a selective PPARG antagonist. The protein expressions of Caspase1 and GSDMD were further confirmed by IF staining. Results showed that RES pretreatment significantly reduced the protein intensities of Caspase1 and GSDMD, which were blocked by GW9662 (*p* < 0.05, Figure 5G,H), suggesting that PPARG is involved in inflammation and pyroptosis regulation.

### 3.6. Resveratrol Alleviates the Effect of LPS on the Steroidogenesis of Goat GCs by Activating PPARG

**Figure 6:**
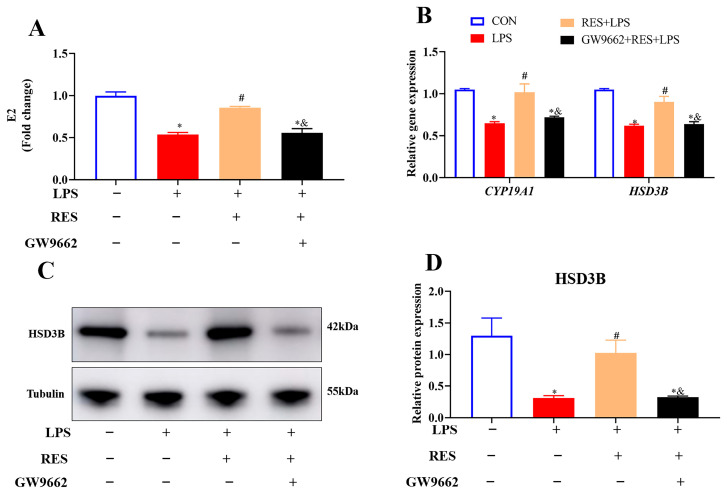
Resveratrol alleviates the effect of LPS on hormone synthesis by activating PPARG in goat GCs. Cells were treated with or without GW9662 (1 μM for 12 h), RES (1 μM for 6 h) or LPS (4 μg/mL for 12 h). (**A**) E2 content; (**B**) relative mRNA abundances of *CYP19A1* and *HSD3B*; (**C**) representative protein bands of HSD3B; (**D**) relative protein abundances of HSD3B. All data are presented as the mean ± SEM. * indicates *p* < 0.05 compared with CON group. # indicates *p* < 0.05 compared with LPS group. & indicates *p* < 0.05 compared with RES + LPS group.

To investigate whether RES alleviates the effect of LPS on the steroidogenesis of goat GCs through PPARG, GCs were assigned to four groups to detect the E2 level and steroidogenic gene and protein expression. Results showed that compared with the LPS group, RES pretreatment increased the content of E2 (*p* < 0.05, Figure 6A) and the mRNA abundances of *CYP19A1* and *HSD3B*, as well as HSD3B protein expression (*p* < 0.05, Figure 6B–D), which were all blocked by GW9662.

### 3.7. Resveratrol Alleviates the Effect of LPS on Goat GCs by Activating the PPARG/NRF2/HO-1 Signaling Pathway

**Figure 7:**
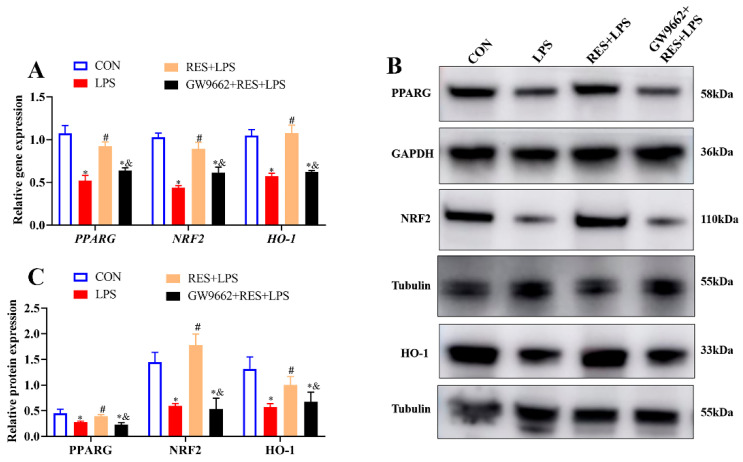
Resveratrol alleviates the effect of LPS on GCs by activating PPARG/NRF2/HO-1 pathway. Cells were treated with or without GW9662 (1 μM for 12 h), RES (1 μM for 6 h), or LPS (4 μg/mL for 12 h). (**A**) Relative mRNA abundances of PPARG, *NRF2*, and *HO-1*; (**B**) representative protein bands of PPARG, NRF2, and HO-1; (**C**) relative protein abundances of PPARG, NRF2, and HO-1. All data are presented as the mean ± SEM. * indicates *p* < 0.05 compared with CON group. # indicates *p* < 0.05 compared with LPS group. & indicates *p* < 0.05 compared with RES + LPS group.

To explore the possible regulatory mechanism of RES, the expression levels of PPARG, NRF2, and HO-1 were measured. Pretreatment with RES significantly increased the gene and protein expressions of PPARG, NRF2, and HO-1 compared with the LPS group, while GW9662 significantly inhibited these upregulations (*p* < 0.05, Figure 7A–C).

## 4. Discussion

### 4.1. Effects of Resveratrol on LPS-Induced Oxidative Stress, Inflammation, and Pyroptosis in Goat Granulosa Cells

In the current study, we found that RES alleviates the effect of LPS on oxidative stress, inflammation, pyroptosis, and estrogen synthesis in GCs by activating the PPARG/NRF2/HO-1 signaling pathway.

To establish the GCs inflammation model, cells were exposed to different concentrations and times of LPS. LPS at 4 μg/mL for 12 h increased the gene expressions of IL-1β, IL-6, and TNF-α, as well as GSDMD, Caspase1, and NLRP3. Previous studies have shown that exposure to LPS at 1 μg/mL for 24 h caused endoplasmic reticulum stress by increasing the expression of the above inflammatory genes in mouse GCs [41], LPS at 0.1 μg/mL for 24 h increased the gene expressions of IL-1β and TNF-α in cow GCs [42], and LPS at 10 μM for 24 h increased the expression of NLRP3, caspase1, and GSDMD in human granulosa-like tumor cell lines [43]. Therefore, the different dosage and time effects of LPS on GCs might be due to the animal species.

To investigate the rescue effect of RES on LPS in GCs, the concentrations and times were screened by detecting inflammation and pyroptosis indicators, and pretreatment with RES at 1 μM for 6 h reduced the expressions of IL-1β, IL-6, TNF-α, GSDMD, Caspase1, and NLRP3. Previous studies have shown that RES at 20 or 100 μM for 24 h protected luteinized GCs from hydrogen peroxide in rat ovaries [44]. RES at 5 μM for 24 h inhibited NLRP3 inflammasome activation and macrophage pyroptosis by increasing the expressions of TNF-α, IL-1β, and IL-6 in mice GCs [45]. RES at 30, 15, and 7.5 μM inhibited the activation of NLRP3 inflammasomes and pyroptosis of macrophages by reducing the levels of IL-1β and Caspase1 in rats exposed to monosodium urate [46]. Therefore, the effect of RES dosage and time on inflammation and pyroptosis might depend on cell types or animal species.

To investigate the mechanism of RES alleviating the impact of LPS on GCs, GW9662 (an antagonist of PPARG) was applied. It was found that GW9662 at 1 μM for 12 h blocked the effect of RES on the inflammatory (IL-1β, IL-6, TNF-α) and pyroptosis (GSDMD, Caspase1, and NLRP3) gene expressions. It was reported that Berberine upregulated PPARG to inhibit the expressions of NLPR3, Caspase1, and GSDMD, which can be blocked by GW9662 (10 μM for 6 h) in mice [47]. Salvianolactone acid A partially alleviated an acute lung injury by reducing the expression of NLRP3 and IL-1β, which was blocked by GW9662 (1 μM for 24 h) [16]. Therefore, the effect of GW9662 treatment concentration and time might be different in various cell types.

The specific concentration and duration of the drugs were then applied to investigate the effect and mechanism of RES that alleviated the effect of LPS in goat GCs. The current increase in MDA content, as well as decreases in SOD activity and T-AOC content induced by LPS, were consistent with the findings of a study in mice that demonstrated that LPS can increase MDA content and decrease the SOD activity and T-AOC level [48]. The present alleviation effect of RES on the alterations of the SOD activity, as well as MDA and T-AOC content induced by LPS, was consistent with that in a mouse diabetic retinopathy model [49], and in fishes exposed to ammonia [50]. Furthermore, GW9662 blocked the alleviation of RES on the LPS in oxidative indexes, which is consistent with the study by Liu et al., who found that GW9662 protected mice from hepatic ischemia/reperfusion injury by aggravating oxidative stress [51]. All these indicate that RES can alleviate the effect of LPS on oxidative stress in goat GCs by activating the PPARG signal.

LPS can cause ROS production, which can damage the structure and function of cell membranes [52]. RES can directly react with multiple ROS free radicals, reducing oxidative damage to intracellular biological macromolecules [53]. Moreira-Pinto et al. [54] treated human GCs with different RES concentrations for different durations and found that low doses of RES could alleviate ROS production. The current increase in ROS, induced by LPS in goat GCs, was reduced by RES, which was blocked by GW9662; this is consistent with the study that GW9662 blocked the effect of Astragaloside IV-induced PPARG in intestinal epithelial cells and reduction in ROS production [55]. Therefore, RES can alleviate the effect of LPS on the ROS content in goat GCs by activating the PPARG signal.

The current LPS-stimulated expressions of IL-1β, TNF-α, and IL-6, reduced by RES, which were blocked by GW9662, are consistent with previous studies. Chen et al. found that LPS enhanced the expressions of IL-1β, TNF-α, and IL-6 in neonates with sepsis [56]. RES induced a dose-dependent inhibition of IL-1β, IL-6, and TNF-α production in T cells [57]. RES also reduced the expression of IL-6, IL-1β, and TNF-α induced by Mycoplasma gallisepticum, both in vivo and in vitro [58]. GW9662 reversed the protective effect of docosahexaenoic acid on bovine mammary epithelial cells exposed to LPS, and increased the expressions of TNF-α, IL-6, and IL-1β [59]. Taken together, RES alleviates the effect of LPS on the inflammation of goat GCs by activating the PPARG signal.

The current LPS-stimulated expressions of NLRP3, Caspase1, and GSDMD were reversed by RES and were blocked by GW9662, which is consistent with previous studies. Wei et al. found that LPS increased cell membrane permeability mediated by GSDMD, resulting in pyroptosis in rats [60]. Luo et al. reported that bergapten inhibits NLRP3 inflammasome activation and LPS-induced pyroptosis in rat J774A.1 cell line [61]. Consistent with our findings, RES improved gouty arthritis, which might inhibit NLRP3 inflammasomes in rats [62]. Magnolol reduced the GSDMD gene expression through PPARG; however, GW9662 could block the reduction [63]. Taken together, RES can alleviate the effect of LPS on pyroptosis in goat GCs by activating the PPARG signal.

HSD3B is a steroid-metabolizing enzyme that is widely present in the synthetic pathways of steroid hormones and can catalyze the transformation of various steroid substrates [64]. CYP19A1 is a member of the cytochrome P450 superfamily, and a key enzyme involved in estrogen synthesis [65]. The current reduction in estrogen secretion accompanied by HSD3B and CYP19A1 expression was alleviated by RES, which was blocked by GW9662. Consistent with our findings, LPS reduced CYP19A1 expression in bovine GCs from large follicles and luteal HSD3B expression in cattle and cows [66]. RES treatment increased the level of HSD3B in Leydig cell lines in mice [67], alleviated high-glucose-induced inhibition of steroidogenesis and the expressions of CYP19A1 and HSD3B in mouse GCs [68]. GW9662 reduces PPARG and HSD3B expression in human GCs [69]. All these indicate that RES can alleviate the effect of LPS on hormone synthesis in goat GCs by activating the PPARG signal.

### 4.2. Involvement of the PPARG/NRF2/HO-1 Pathway and Physiological Implications

Furthermore, the PPARG signaling pathway, which is involved in the regulation of cellular oxidative stress, inflammation, and pyroptosis, was explored. Previous studies have demonstrated that RES can upregulate PPARG expression in mouse MIN6 cells [70] and regulate the expressions of NRF2 and HO-1 expression in human ovaries [31]. All these studies are consistent with our current findings, which demonstrate that RES alleviates the LPS-induced reduction in PPARG/NRF2/HO-1 expression. When GW9662 was added, the expression of PPARG decreased, and the expressions of NRF2 and HO-1 also declined in mouse hearts [71]. This is consistent with the current inhibition by GW9662 in the expression of PPARG mediated by RES, as well as the expression of its downstream genes. Given that PPARG inhibitors have an impact on estrogen synthesis, oxidative stress, inflammation, and pyroptosis, it is possible that they exert regulation through the PPARG/NRF2/HO-1 pathway.

Despite the current results showing that RES reduces the oxidative stress and pyroptosis caused by LPS through the PPARG/NRF2/HO-1 signaling pathway, the molecular process of intracellular penetration and receptor engagement of RES is yet to be elucidated. The literature suggests that RES is able to interact with the ligand-binding domain of PPARG and increase the transcriptional regulation of antioxidant and anti-inflammatory genes [20,31]. It is also possible that RES can regulate mitochondrial and endoplasmic-reticulum homeostasis, which results in lowering the accumulation of ROS and the activation of inflammasomes. In order to understand these mechanisms in goat GCs, further studies through receptor-binding and molecular-docking techniques are justified. A limitation of this study is that the reproductive stage of the animals could not be determined, because ovarian samples were collected post-slaughter, without hormonal or ultrasonographic evaluation. Future studies should include reproductive-cycle assessment to minimize physiological variation.

## 5. Conclusions

The present study proved that LPS exposure caused significant oxidative stress, inflammation, and pyroptosis, leading to decreased estrogen synthesis in goat granulosa cells. Resveratrol (RES) treatment effectively reversed these effects by increasing antioxidant enzyme activities, reducing reactive oxygen species and inflammatory cytokine levels, and improving steroidogenic gene expression. These protective effects were mediated through the activation of the PPARG/NRF2/HO-1 signaling pathway, as confirmed by the inhibitory effects of GW9662 on RES-induced protection.

Overall, this work provides clear mechanistic evidence that RES mitigates LPS-induced oxidative and inflammatory damage in granulosa cells through PPARG/NRF2/HO-1 activation. These findings suggest that RES could serve as a potential nutritional or therapeutic approach to protect ovarian function and fertility against inflammation-related reproductive disorders in ruminants.

## Figures and Tables

**Table 1:** Primer sequences used for RT-qPCR. All primers were amplified at a uniform annealing temperature of 60 °C.

Gene	Gene ID	Primer Sequence (5′-3′)	Product Length/bp
*CAT*	XM_005690077.3	F: CATTACCAGATACTCCAAGGCGAAGG	234
		R: TGGCTATGGATAAAGGACGGAAACAG	
*Caspase1*	NC_030822.1	F: TATGCCTGGTCCTGTGACCT	102
		R: AGTCACTCTTTCAGCGGTGG	
*CYP19A1*	NM_001285747.1	F: CAGCATGGTGTCCGAAGTTG	133
		R: GGGCCCAATTCCCAGAAAGT	
*GAPDH*	NM_001034034.1	F: CGACTTCAACAGCGACACTCAC	119
		R: CCCTGTTGCTGTAGCCGAATTC	
*GSDMD*	NC_030821.1	F: GTTATTGGCTCTGACTGGG	120
		R: GAAGCACGAACGTGGATG	
*HO-1*	NM_001285567.1	F: CGGCAGCAAGGCACAAGACTC	97
		R: GAGGACCCATCGCAGGAGAGG	
*HSD3B*	NM_001285716.1	F: AGACCAGAAGTTCGGGAGGAA	292
		R: TCTCCCTGTAGGAGTTGGGC	
*IL-1β*	XM-013967700.2	F: CCACCTCCTCTCACAGGAAATG	100
		R: GATACCCAAGGCCACAGGAATC	
*IL-6*	NM-001285640.1	F: TACCTGGACTTCCTCCAGAAC	245
		R: CGAATAGCTCTCAGGCTGAAC	
*NLRP3*	NC_030814.1	F: CCGTCTGGGTGAGAGCGTGAA	78
		R: TCCTGTTGGCTCCTGTGTTCCT	
*NRF2*	NM_001314327.1	F: GCCCAGTCTTCAATGCTCCTTCTC	113
		R: TTCCTCCCAAACTTGCTCAATGTCC	
*PPARG*	NM_001285658.1	F: ATATCCCCGGCTTCGTGAAC	210
		R: CAGCAAACTCGAACTTGGGC	
*SOD*	XM_018053428.1	F: CGGCCTACGTGAACAACCTCAAC	261
		R: GGACACCAACAGATACAGCAGTCAG	
*TNF-a*	NM 001286442.1	F: CCACGTTGTAGCCAACATCAG	134
		R: AGATGAGGTAAAGCCCGTCAG	

**Table 2:** Details of antibodies for WB.

Antibodies	Cat No.	Supplier	Dilution	Host	Antigen Source
Primary antibody					
Caspase1	A19792	Abclonal	1:1000	Rabbit	Human
GSDMD	A17308	Abclonal	1:1000	Rabbit	Human
GAPDH	A19056	Abclonal	1:1000	Rabbit	Human
HO-1	A21452	Abclonal	1:1000	Rabbit	Human
IL-1β	A16288	Abclonal	1:1000	Rabbit	Human
NRF2	A11159	Abclonal	1:1000	Rabbit	Human
PPARG	A0270	Abclonal	1:1000	Rabbit	Human
SOD	24127-1-AP	Proteintech	1:1000	Rabbit	Human
Tubulin	AF7011	Affinity	1:2000	Rabbit	Human
Secondary antibody					
HRP anti-rabbit IgG	AS014	Abclonal	1:5000	Goat	Human

## Data Availability

Data will be made available on request.

## Abbreviations

The following abbreviations are used in this manuscript:

Caspase1	Cysteine–aspartic acid protease 1
CCK8	Cell counting kit-8
CYP19A1	Cytochrome P450 family 19 subfamily A member 1
E2	Estradiol
GCs	Granulosa cells
GSDMD	Gasdermin D
GW9662	PPARG antagonist
H&E	Hematoxylin and eosin
HO-1	Heme oxygenase 1
HSD3B	Hydroxysteroid dehydrogenase 3 beta
IF	Immunofluorescence
IL-1β	Interleukin-1β
IL-6	Interleukin-6
LPS	Lipopolysaccharide
MDA	Malondialdehyde
NLRP3	Nucleotide-binding oligomerization domain-like receptor protein 3
NRF2	Nuclear factor erythroid 2-related factor 2
PPARG	Peroxisome proliferator-activated receptor gamma
RIA	Radioimmunoassay
ROS	Reactive oxygen species
RT-qPCR	Real-time quantitative polymerase chain reaction
SOD	Superoxide dismutase
T-AOC	Total antioxidant capacity
TG	Triglycerides
TNF-α	Tumor necrosis factor-alpha
WB	Western blot
